# Benign disease prone to be misdiagnosed as malignant pulmonary nodules: Minute meningothelioid nodules

**DOI:** 10.1111/1759-7714.13061

**Published:** 2019-04-09

**Authors:** Xiao‐Xiao Peng, Li‐Xu Yan, Chao Liu, Si‐Yun Wang, Wen‐Feng Li, Xing Gao, Xue‐Wu Wei, Qing Zhou

**Affiliations:** ^1^ Guangdong Lung Cancer Institute, Guangdong Provincial Key Laboratory of Translational Medicine in Lung Cancer Guangdong Provincial People's Hospital & Guangdong Academy of Medical Sciences Guangzhou China; ^2^ School of Medicine South China University of Technology Guangzhou China; ^3^ Department of Pathology and Laboratory Medicine Guangdong Provincial People's Hospital & Guangdong Academy of Medical Sciences Guangzhou China; ^4^ PET Center Guangdong Provincial People's Hospital & Guangdong Academy of Medical Sciences Guangzhou China

**Keywords:** Lung, malignant pulmonary nodules, minute meningothelioid nodules

## Abstract

**Background:**

The lung is one of the most common target organs for malignant tumor metastasis. The existence of lung metastasis may have a decisive effect on the choice of treatment regimen. Minute pulmonary meningothelial‐like nodules (MPMNs) usually present as ground‐glass opacity or solid nodules, mimicking the imaging findings of malignant pulmonary nodules. This study summarizes the clinical, radiological, and pathological features of MPMNs to distinguish them from malignant pulmonary nodules.

**Methods:**

The Guangdong Lung Cancer Institute Pathology Information System was searched using the key words “minute meningothelioid nodules” and “lung.” Patients who underwent pulmonary resection from 23 February 2009 to 31 May 2017 were included in the study. The 11th edition of *Rosai and Ackerman's Surgical Pathology* was used to confirm the diagnosis. The clinical, imaging, and pathological characteristics of MPMNs were recorded.

**Results:**

Twelve patients had MPMNs. MPMNs were associated with cancerous or precancerous lesions (10/12), female gender (11/12), and non‐smokers (11/12). Four patients were misdiagnosed with malignant pulmonary nodules before surgery. Positron emission tomography‐computed tomography revealed an increased maximum standardized uptake value in one patient. Immunohistochemistry identified eight specimens positive for vimentin, EMA, and PR and negative for TTF‐1 and CK.

**Conclusions:**

MPMNs tend to coexist with malignant tumors, mimicking the imaging findings of malignant pulmonary nodules, thus resulting in misdiagnosis. Dynamic monitoring or an invasive examination may help to distinguish MPMNs from malignant lesions.

## Introduction

The lung is one of the most common target organs for malignant tumor metastasis, occurring, for example, in lung, gastrointestinal, and breast cancers. Previous studies have reported that during autopsy, lung metastases are found in 25–30% of all patients with malignant disease.^1^ The existence of lung metastasis may have a decisive effect on the choice of treatment regimen. In clinical work, imaging technologies are generally used to identify lung metastasis; however, it is difficult to distinguish malignant from benign pulmonary nodules in some patients. Thus, some benign lung nodules are misdiagnosed as lung metastases, resulting in incorrect clinical staging and treatment. Minute pulmonary meningothelial‐like nodules (MPMNs) are a kind of benign pulmonary nodule. They are small lesions, usually incidentally discovered in surgical or autopsy specimens of the lung.^2^

In 1960, Korn *et al.* first named these lesions “pulmonary chemodectoma” because of their microstructure, cytologic characteristics, and relationship with vessels. They were not associated with scarring or any specific pulmonary disease or physiological disturbance.^3^ However, follow‐up studies revealed that instead of containing endocrine granules, they closely resembled meningothelial cells.^4^, ^5^ Therefore, Gaffey *et al.* renamed them “meningothelial‐like nodules.”^6^ The third edition of the World Health Organization International Histological Classification of Tumors categorized these as “minute meningothelioid nodules.” According to a clonal study, subsequent studies have described these nodules as a transition between reactive and neoplastic proliferation.^7^

As for the pathogenesis, two different viewpoints currently exist. On the one hand, Weissferdt *et al.* pointed out that MPMNs share common genetic pathways with central nervous system meningiomas.^8^ On the other hand, several studies have indicated that MPMNs might be associated with chronic lung or serious cardiac diseases, such as thromboemboli, cardiac failure, chronic bronchitis and emphysema, and atypical adenomatous hyperplasia.^9^, ^10^ These diseases may stimulate the lung by stretching or stiffening the alveolar septa, or by causing hypoxia, ischemia, parenchymal destruction, or some combination of these factors, and then induce the formation of MPMNs, which is a nonspecific hyperplastic response to many forms of injury to the pulmonary interstitium.

In recent years, with the increasing use of high‐resolution computed tomography (CT), more MPMNs are occasionally detected during a medical examination or before surgery. Distinguishing MPMNs from malignant hematogenous metastatic lesions contributes to the appropriate clinical management of patients with multiple small pulmonary nodules. Cases such as these were summarized in the present study in order to draw attention to these rare lesions. The records of 12 patients with MPMNs from the Guangdong Lung Cancer Institute database were retrospectively analyzed. The clinical, imaging, and pathological characteristics of MPMNs identified in surgically resected lungs were recorded. A typical case misdiagnosed as malignant pulmonary nodules was analyzed.

## Methods

### Patients and study design

The Guangdong Lung Cancer Institute Pathology Information System was searched using the key words “minute meningothelioid nodules” and “lung.” Patients who underwent pulmonary resection from 23 February 2009 to 31 May 2017 were included in this study. All specimens were obtained from pulmonary resections performed at the Guangdong Lung Cancer Institute/Guangdong Provincial People's Hospital. Patient data were analyzed to summarize the clinical, imaging, and immunohistochemical (IHC) characteristics.

### Confirmation of previous diagnoses

The slides of patients who developed MPMNs were obtained from the slide library to confirm the previous diagnoses. Two senior pathologists blinded to the diagnoses independently rechecked these slides. Any difference in opinion on the diagnosis of these lesions was resolved by discussion.

The diagnostic criteria for MPMNs according to the 11th edition of *Rosai and Ackerman's Surgical Pathology* are as follows: (i) most nodules are < 5 mm in diameter, solitary, well circumscribed, and firm, with a yellowish tan to gray cut surface; (ii) nodules are primarily located in the lung, except for central nervous system meningioma metastasis; (iii) cells resemble meningothelial rather than chemoreceptor cells, according to electron microscopy; and (iv) cells are positive for vimentin, EMA, CK, and other melanoma markers, and negative for neuroendocrine markers.^11^

### Data collection

Clinical information (including age, gender, smoking history, and clinical symptoms), medical history (including coexisting disease, treatment regimen, operation information, and prognosis), imaging features (including CT and positron emission tomography–CT scan images), and pathological features (including hematoxylin–eosin staining and other specific staining results) were obtained from electronic health records.

Non‐small‐cell lung cancer (NSCLC) and esophageal cancer tumor and node staging was based on the results of surgical resection or comprehensive imaging using the seventh edition of the Union for International Cancer Control Tumor Node Metastasis (TNM) Staging System.

## Results

### Patient characteristics

Thirteen patients were diagnosed with MPMNs; a patient with associated central nervous system meningiomas was excluded from this study. Two pathologists consistently diagnosed the remaining 12 patients with MPMNs. The clinical characteristics of these patients are summarized in Tables 1 and S1. The average age of the patients was 54 (range: 32–72) years. The main coexisting diseases in these 12 patients with MPMNs were: lung cancer (7 patients), atypical adenomatous hyperplasia (2 patients), esophageal cancer (1 patient), pulmonary granuloma (1 patient), and tubular adenoma of the colon (1 patient). Female gender (11/12), non‐smoking (11/12), and malignant tumors or precancerous lesions (10/12) were significantly associated with MPMNs.

**Table 1:** Clinical characteristics of patients with MPMNs

Characteristic	Patients with MPMNs
Age (years)	
Mean	54
Range	32–72
Gender	
Male	1
Female	11
Smoking history	
Yes	1
No	11
Resected lobe	
Left	3
Right	9
Main coexisting disease	
Pulmonary adenocarcinoma	4
Squamous cell lung cancer	2
Lymphoid‐like lung cancer	1
Squamous cell esophageal cancer	1
Pulmonary AAH	2
Pulmonary granuloma	1
Tubular adenoma of colon	1

AAH, atypical adenomatous hyperplasia, MPMNs, minute pulmonary meningothelial‐like nodules.

### Radiological characteristics of minute pulmonary meningothelial‐like nodules (MPMNs)

**Figure 1:**
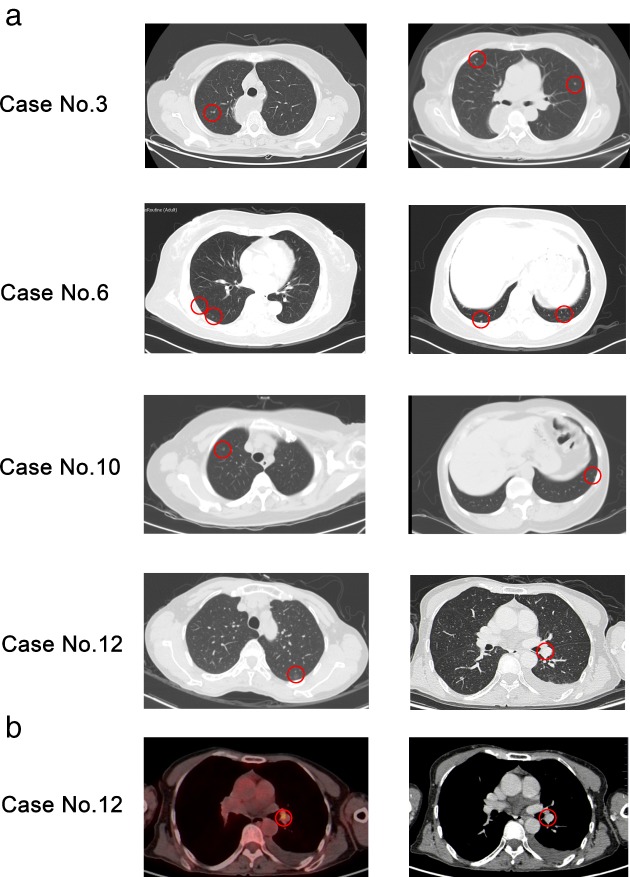
(**a**) Thin‐section chest computed tomography scan (1 mm collimation) demonstrated multiple well‐defined nodules of various sizes in four patients. (**b**) Preoperative 18‐fluoro‐2‐deoxy‐D‐glucose positron emission tomography examination revealed an increased maximum standardized uptake value of 4.8 in one patient. Red circles indicate pathological representation of minute pulmonary meningothelial‐like nodules.

MPMNs were found in 4 of the 12 patients via CT scans and were misdiagnosed as malignant nodules. The CT images are shown in Figure 1a. The nodules usually presented as randomly distributed, rounded, multiple solid nodules or ground‐glass opacities (GGOs), were well defined, < 5 mm in diameter, and mimicked primary lung or hematogenous metastatic tumors. No spicule, pleural indentation, or other malignant signs were observed. After at least six months of preoperative or postoperative dynamic CT monitoring, no changes in the lesions were observed. Among these four patients, one underwent preoperative 18‐fluoro‐2‐deoxy‐D‐glucose positron emission tomography examination, and the nodule's maximum standardized uptake value (SUVmax) was 4.8 (Fig 1b), which was highly suspicious of malignancy and made it even more difficult to differentiate MPMNs from malignant pulmonary nodules.

### Pathological characteristics of MPMNs

**Figure 2:**
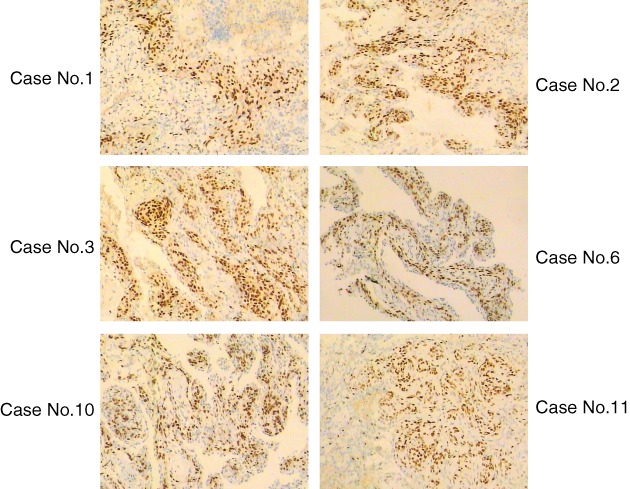
PR staining of all minute pulmonary meningothelial‐like nodules after immunohistochemical staining.

The detailed IHC results are shown in Table 2. Eight specimens tested positive for vimentin, EMA, and PR and negative for TTF‐1 and CK. PR staining of all IHC stained MPMNs is shown in Figure 2.

**Table 2:** Results of immunohistochemical analyses

	Case no.											
	1	2	3	4	5	6	7	8	9	10	11	12
Gender	F	F	F	F	F	F	F	F	F	F	M	F
Age (y)	72	54	63	37	56	68	32	54	46	54	53	59
Maximum lesion diameter (mm)	3	1.5	3	< 1	2	5	1	1	1	4	1	5
EMA	++	++	++	++	+	+++	NE	NE	NE	++	+++	NE
VIM	NE	+++	+++	+++	+++	+++	NE	NE	NE	+++	+++	NE
PR	+++	+++	+++	NE	NE	+++	NE	NE	NE	+++	+++	NE
TTF‐1	NE	–	–	NE	NE	NE	NE	NE	NE	NE	NE	NE
CK	NE	NE	–	NE	–	NE	NE	NE	NE	NE	NE	NE
Ki67	NE	NE	1%+	NE	< 1%+	Only individual cells+	NE	NE	NE	< 1%+	NE	NE

–, No staining; +, faint staining; ++, obvious staining; +++, dense staining;

NE, not evaluated; VIM, vimentin.

### Characteristics of diagnosis and treatment (typical cases)

All of the MPMNs were discovered incidentally: eight in surgical lung specimens, and four by CT scan subsequently confirmed by pathological examination. MPMNs were the primary target for surgical resection in the four patients detected via CT scan. Although these nodules were all found accidentally, they coexisted with other suspected malignant diseases.

**Figure 3:**
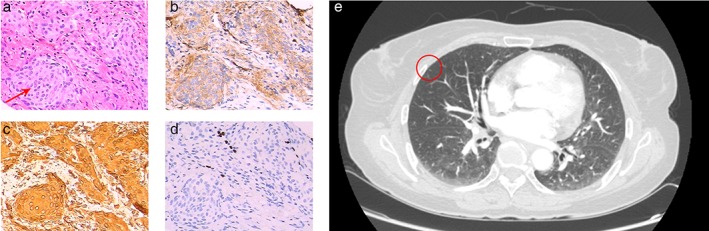
(**a**) In case no. 3, multiple foci were found in the removed lobe, the largest being 3 mm. Although they did not have encapsulation, they were typically well demarcated and composed of mild spindle cells, which were arranged in a swirl. The cells had no obvious atypia, and no mitotic figures were found (arrowhead) (hematoxylin and eosin stain, original magnification ×200). On immunohistochemical staining, the cells were (**b**) positive for EMA (obvious staining) and (**c**) vimentin (dense staining), and (**d**) negative for TTF‐1. (**e**) Computed tomography appearance of the corresponding pulmonary nodule (red circle).

Case no. 3 was a 63‐year‐old woman diagnosed with cT3N1M1 stage IV squamous cell carcinoma of the lower thoracic esophagus (bilateral lung metastases). After three cycles of chemotherapy, an enhanced CT showed no change in the multiple tiny nodules in her bilateral lung. Her doctor‐in‐charge discovered this situation and doubted whether they were actually lung metastases. A lung biopsy was suggested, but was rejected by the patient and her family; instead they urged for radical resection of the esophageal carcinoma and wedge resection of the right middle lobe. Postoperative pathology proved that these small opacities were MPMNs (Fig 3). After eight months, no change was observed in the remaining opacities.

In case no. 6, the main lesions were multiple pulmonary GGOs and multiple hepatic nodules. In case no. 10, the multiple GGOs coexisted with an 8 mm pulmonary cavitary lesion. These dispersed foci on the CT scan implied hematogenous metastases. Both patients underwent surgical resection to confirm whether these were tumor metastases and to identify the histopathological type. The pulmonary GGOs in both cases were confirmed to be MPMNs, and the cavitary lesion in case no. 10 was a granuloma. No change was observed in the remaining pulmonary and liver lesions after six months.

## Discussion

Previous studies have mainly focused on the pathology, pathogenesis, and clonal study of MPMNs, and only a few case reports have analyzed their clinical significance. The present study is a rare summary with detailed clinical data analyzing how MPMNs affect the diagnosis of a disease. According to previous studies, the frequency of MPMNs in autopsy‐based analyses ranges from 0.3 to 0.5%, while the frequency in surgical‐based analyses ranges from 1.1 to 9.5%.^10^, ^12^, ^13^ These results were influenced by the different numbers of random histological sections studied and whether the pathologist sorted these sections carefully. A few studies have reported that the average size of MPMNs is 1.5 mm (range: 0.3–6.0 mm).^14^, ^15^ Our results were consistent with these previous findings. MPMNs are more often found in patients with malignant pulmonary tumors than in those with benign diseases (7.3 vs. 2.5%; *P* = 0.044). In particular, they more often occur in patients with lung adenocarcinoma than in those with other primary pulmonary malignant tumors (9.4 vs. 4.5%, odds ratio 2.33, 95% confidence interval 1.35, 4.02; *P* < 0.01).^11^ As for gender, conventional studies have reported that MPMNs are more frequently detected in women (range: 65–100%) than in men.^3^, ^8^, ^10^, ^12^ Only 12 patients with MPMNs were located in our database, which may be attributed to omission or misdiagnosis of this disease. If more attention is paid to this disease, the discovery rate may increase. In addition, these cases were associated with cancerous or precancerous lesions (10/12), female gender (11/12), and non‐smoking (11/12), in line with the results of previous studies.

The tiny size of MPMNs and their association with other pulmonary pathological changes have led to a lack of description in radiology studies. Because of the low specificity in an imaging diagnosis for patients with MPMNs, even the maximum standardized uptake value may increase, making it almost impossible to distinguish them from malignant pulmonary nodules through a single‐image examination. According to our results and those of previous studies, MPMNs do not grow during follow‐up for at least six months. Although they are similar in appearance to malignant nodules, they grow in a benign manner. Therefore, the possibility of MPMNs must be considered for lesions that are suspected to be multiple metastatic pulmonary nodules but do not change after long‐term monitoring or even systemic antitumor treatment. In‐depth texture analysis or dynamic observation of imaging may help to distinguish them from malignant pulmonary nodules because MPMNs do not exhibit any change for a long period of time.

As early as 1999, an IHC analysis revealed that approximately half of all MPMNs are PR positive.^12^ In 2002, Pelosi *et al.* also reported that MPMNs in six patients exhibited immunoreactivity for PR.^13^ In this study, six patients tested positive for PR by IHC. In 2005, Ishibashi *et al.* reported that 56.3% of patients with pulmonary adenocarcinoma and 61.9% of female patients with NSCLC tested positive for PR.^16^ These results indicate that progesterone stimulation might contribute to the occurrence of pulmonary adenocarcinoma and MPMNs, finally resulting in the frequent co‐occurrence of these two conditions in female patients with adenocarcinoma.

Of the 12 patients with MPMNs in this study, eight were discovered in surgical specimens of the lung, while the other four were discovered by CT scan and confirmed by pathological examination. Fortunately, the patients in this study underwent surgery and the lung nodules were not found to be malignant. However, this indicates that more such misdiagnosed cases might exist that have not been discovered. Only two cases of misdiagnosis have been reported in previous studies.^17^, ^18^ Jean Picquet *et al.* reported a case of an MPMN that presented as a solitary subpleural pulmonary nodule in a patient with breast cancer. The absence of radiographic changes after six months of chemotherapy led to resection of the breast and lung lesions. The postoperative diagnoses were early‐stage breast cancer and MPMN. After six months, the patient presented no evidence of tumor recurrence.^17^ Therefore, we make the following suggestions to reduce the misdiagnosis rate. If a patient has a malignant tumor but lung metastases are only found via CT scan, confirmation of lung metastasis should be made. This should determine whether the malignant disease has developed into an advanced stage. If a patient is diagnosed with an advanced malignant tumor and has been administered systematic treatment but no change is observed in the nodules, surgery or biopsy should be considered to confirm the characteristics.

This study has a few limitations. This was a retrospective study and cases were retrieved from the pathology system and electronic health records instead of rechecking the slides of patients who underwent pulmonary resection. Hence, some MPMNs could have been overlooked. The limited number of cases was insufficient to determine the epidemiological features.

In conclusion, MPMNs are a kind of benign disease prone to misdiagnosis as malignant pulmonary nodules. Their association with malignant tumors and similarity to malignant pulmonary nodules in imaging make distinction even more difficult. Dynamic monitoring or an invasive examination may help to distinguish MPMNs from malignant lesions.

## Disclosure

No authors report any no conflict of interest.

## Supporting information


**Table S1.** Detailed clinical information of the 12 patients with minute pulmonary meningothelial‐like nodules (MPMNs) including gender, age, smoking index, symptoms, resected lobe, whether the lesion is visible on computed tomography scan, and main coexisting disease.
