# Supplementary material for: Benign disease prone to be misdiagnosed as malignant pulmonary nodules: Minute meningothelioid nodules
Source: Thorac Cancer. 2019 Apr 9;10(5):1182–7. doi: 10.1111/1759-7714.13061 (PMC6501004; doi:10.1111/1759-7714.13061)
Supplement: Supplementary file 1 — Table S1. Detailed clinical information of the 12 patients with minute pulmonary meningothelial‐like nodules (MPMNs) including gender, age, smoking index, symptoms, resected lobe, whether the lesion is visible on computed tomography scan, and main coexisting disease. [file TCA-10-1182-s001.docx]

**Supplementary table 1.** Detailed clinical information of the 12 MPMNs patients including sex, age, smoking index, symptoms, resected lobe, whether the lesion is visible on CT scan and main coexisting disease.

| Case number | Sex | Age  (year) | Smoking index | Sympto-ms | Resected lobe | Whether visible on CT scan | Main coexisting disease |
| --- | --- | --- | --- | --- | --- | --- | --- |
| 1 | F | 72 | No | No | Right lower lobe | N | Squamous cell carcinoma in the right lower lobe pT2aN0M0, Stage IB (7th) |
| 2 | F | 54 | No | No | Right upper lobe | N | Adenocarcinoma in the right upper lobe pT1bN0M0, Stage IA (7th) |
| 3 | F | 63 | No | No | Right middle lobe | Y | Squamous cell carcinoma of lower thoracic esophagus pT3N1M0, stage III |
| 4 | F | 37 | No | No | Right upper lobe | N | Lymphoepithelioma-like carcinoma in the right upper lobe pT1bN0M0, Stage IA (7th) |
| 5 | F | 56 | No | No | Right middle lobe | N | Adenocarcinoma in the right middle lobe pT1aN0M0, Stage Ia (7th) |
| 6 | F | 68 | No | No | Right upper lobe | Y | Tubular adenoma of colon |
| 7 | F | 32 | No | No | Left lower lobe | N | Atypical AAH in the left lower lobe |
| 8 | F | 54 | No | Dizziness, Blurred vision | Right upper lobe | N | Peripheral adenocarcinoma in the right upper lobe cT1aN0M1b(brain), Stage IVA (7th) |
| 9 | F | 46 | No | No | Right upper lobe | N | Squamous cell carcinoma in the right upper lobe pT2aN1M0, Stage IIa (7th) |
| 10 | F | 54 | No | No | Left lower lobe | Y | Pulmonary granuloma in the left lower lobe |
| 11 | M | 53 | 900 | No | Right upper and middle lobe | N | Squamous cell carcinoma in the right upper lobe pT2aN1M0, Stage IIa (7th) |
| 12 | F | 59 | No | Cough | Left upper lobe and left lower lobe | Y | AAH in the left lower lobe |

AAH, Atypical adenomatous hyperplasia.
